# Prediction of atmospheric PM_2.5_ level by machine learning techniques in Isfahan, Iran

**DOI:** 10.1038/s41598-024-52617-z

**Published:** 2024-01-24

**Authors:** Farzaneh Mohammadi, Hakimeh Teiri, Yaghoub Hajizadeh, Ali Abdolahnejad, Afshin Ebrahimi

**Affiliations:** 1 Environment Research Center, Research Institute for Primordial Prevention of Non-Communicable Diseases, Isfahan University of Medical Sciences, Hezar Jerib Street, Isfahan, 8174673461 Iran; 2 Department of Environmental Health Engineering, Faculty of Health, Isfahan University of Medical Sciences, Isfahan, Iran; 3 Department of Environmental Health Engineering, School of Public Health, Maragheh University of Medical Sciences, Maragheh, Iran

**Keywords:** Climate sciences, Environmental sciences, Environmental social sciences, Mathematics and computing

## Abstract

With increasing levels of air pollution, air quality prediction has attracted more attention. Mathematical models are being developed by researchers to achieve precise predictions. Monitoring and prediction of atmospheric PM_2.5_ levels, as a predominant pollutant, is essential in emission mitigation programs. In this study, meteorological datasets from 9 years in Isfahan city, a large metropolis of Iran, were applied to predict the PM_2.5_ levels, using four machine learning algorithms including Artificial Neural |Networks (ANNs), K-Nearest-Neighbors (KNN), Support Vector |Machines (SVMs) and ensembles of classification trees Random Forest (RF). The data from 7 air quality monitoring stations located in Isfahan City were taken into consideration. The Confusion Matrix and Cross-Entropy Loss were used to analyze the performance of classification models. Several parameters, including sensitivity, specificity, accuracy, F1 score, precision, and the area under the curve (AUC), are computed to assess model performance. Finally, by introducing the predicted data for 2020 into ArcGIS software and using the IDW (Inverse Distance Weighting) method, interpolation was conducted for the area of Isfahan city and the pollution map was illustrated for each month of the year. The results showed that, based on the accuracy percentage, the ANN model has a better performance (90.1%) in predicting PM_2.5_ grades compared to the other models for the applied meteorological dataset, followed by RF (86.1%), SVM (84.6%) and KNN (82.2%) models, respectively. Therefore, ANN modelling provides a feasible procedure for the managerial planning of air pollution control.

## Introduction

In recent decades, with the fast-growing industries and increasing traffic in the urban areas in Iran, megacities like Isfahan are facing serious air pollution problems. PM_2.5_, particulate matter with an aerodynamic diameter of less than 2.5 µm, as one of the predominant pollutants in the air, has attracted wide attention. PM_2.5_ consists of toxic and hazardous substances that are highly active, leading to its long residence time in the atmosphere and far transportation distance. As a result, exposure to high concentrations of PM_2.5_ has been found to exacerbate the development of cardiovascular and respiratory diseases^1,2^.

To improve air quality management, the Isfahan Department of the Environment has been publishing the daily Air Quality Index (AQI) since 2008. It is worth noting that PM_2.5_ is always the only responsible pollutant for the AQI in Isfahan. Iran has established national ambient air quality standards for PM_2.5_, which are based on EPA standards. These standards include a primary annual mean concentration of 12 µg/m^3^, a secondary annual mean concentration of 15 µg/m^3^, and a 24-h average concentration of 35 µg/m^3^ (source: https://www.epa.gov/criteria-air-pollutants/naaqs-table).

Apart from the pollutant emissions from various sources, the meteorological condition and their interaction greatly influence the level of PM_2.5_. Meteorological factors have high impacts on the diffusion, dilution and deposition of particulate matter. Sometimes, the impact of meteorological conditions on PM_2.5_ accumulations in the lower layer of the atmosphere can be more considerable. It has been reported that lower emissions of the pollutant with undesirable weather conditions can lead to higher PM_2.5_ concentrations compared to higher emissions of the pollutant with desirable weather conditions^3^. For instance, the spatio-temporal variation of wind speed impacts the distribution of PM_2.5_ and its precursors. Factors like wind speed, direction, and stability influence the dispersion and transport of pollutants, ultimately determining their concentration in different areas over time^4^.

The adverse effects of PM_2.5_ on health have prompted researchers to consider the urgent need to simulate and forecast its concentration. In the last decade, many models have been proposed to predict the level of PM_2.5_ in outdoor air. The investigated model's accuracy and skills were amended gently by the scientific advances in numerical, statistical and computational techniques^5^.

The commonly used models for predicting PM_2.5_ concentrations are classical diffusion models, which include the street canyon model and the Gaussian plume model. However, the performance of these models relies on empirical assumptions, and the data and environmental needs are specific to different geographic regions. Therefore, while these models can be applied globally, certain unique requirements may pose challenges, especially when it comes to large-scale applications^6^. Due to the complexity of the atmospheric system, the classical models need a lot of simplification and their expansion to large areas is not very precise. In various studies, diverse machine learning methods, including linear regression, random forest, and deep neural networks, have been employed to forecast PM_2.5_ concentration using aerosol optical depth (AOD) data. By leveraging the correlation between AOD measurements and PM_2.5_ levels, these techniques accurately estimate the levels of air pollution^7^.

In the field of earth science, geographical information systems (GIS) have been widely utilized as a means to store, retrieve, analyze, and visually present spatial data. Statistical methods were initially developed to evaluate multiple parameters, but understanding the interdependencies between variables has always been a primary concern. Consequently, artificial intelligence (AI) models were investigated to examine their potential to assist the analysis of spatial data. Successful handling of spatial data was achieved by the combination of AI models with a GIS^8,9^.

Among the most widely used AI techniques are artificial neural networks (ANNs), K-Nearest-Neighbors (KNN), support vector machines (SVMs) and ensembles of classification trees such as random forest (RF)^10^. Published articles in the field of PM_2.5_ concentration modelling reported the use of various models such as multiple linear regression^11^, artificial neural network^12^, deep convolutional neural networks^13^ Support vector machine^3^ Adaptive Neuro-Fuzzy Inference System (ANFIS)^14^. However, there is a lack of studies that compared the different models' performance and their combination with GIS.

By merging machine learning models and GIS models, the precision of PM_2.5_ level predictions is refined through the utilization of spatiotemporal data. This cutting-edge method provides valuable information on the dispersion and potential consequences of fine particulate matter, thereby assisting in improved management of air quality and initiatives for public health. Therefore, for this research, Isfahan City was chosen as a pilot to forecast the PM_2.5_ level in the air by employing a blend of machine learning models and GIS models. Isfahan metropolis is located in the centre of Iran, Middle East, and categorized as a semi-arid area (temperature range from − 2.4 °C to 36.4 °C), with an annual precipitation of 125–160 mm, relative humidity of 23–60%, and monthly average wind speed of 2.6–8.3 mph. This city usually experiences high air pollution on many days of the year and PM_2.5_ is the main predictor of the air quality index (AQI). Meteorological information and PM_2.5_ data from all sites from 2011 to 2019 were received from the Isfahan Meteorological Administration and the Isfahan Department of Environment, respectively. ANN, KNN, SVM and RF models were evaluated and the best model was selected. After the determination of the best model, the meteorological data of the city for 2020 was applied and by combining the optimal model and GIS, a pollution map was illustrated in each month of 2020 for Isfahan city.

## Material and method

### Monitoring locations

**Figure 1:**
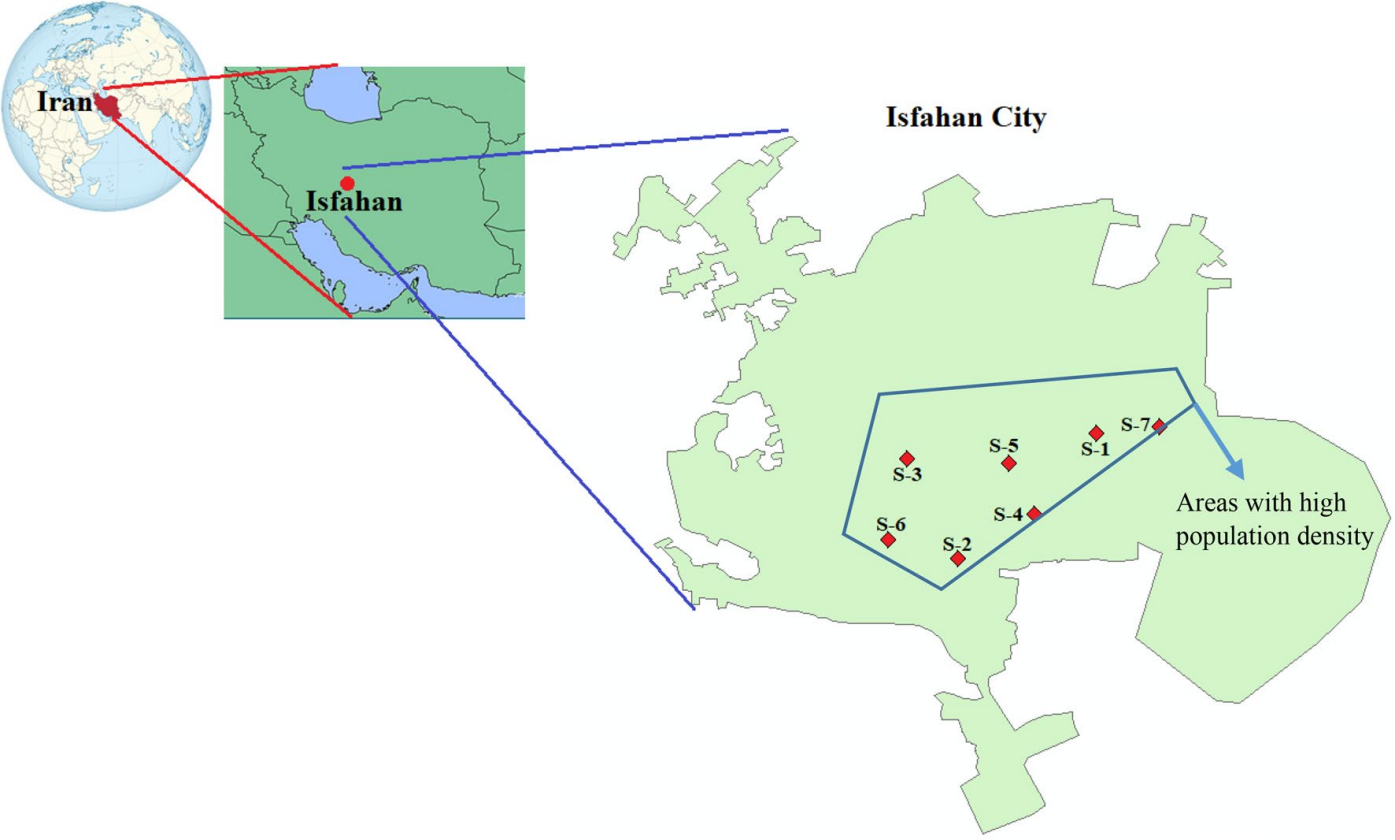
The geographical location of Isfahan City, the selected seven monitoring sites, and the areas with high population density of the studied city.

Isfahan, located in central Iran, is one of the most populous and industrialized cities with approximately 2 million residents and an average population density of 3560 people per km^2^. However, Isfahan has been grappling with a significant air pollution issue. The geographical location of Isfahan province and the selected 7 monitoring sites are shown in Fig. 1, including, Site 1 (32.664 N, 51.702 E), Site 2 (32.622 N, 51.660 E), Site 3 (32.656 N, 51.643 E), Site 4 (32.638 N, 51.684 E), Site 5 (32.655 N, 51.675 E), Site 6 (32.629 N, 51.637 E) and Site 7 (32.667 N, 51.720 E). The monitoring stations are mainly located in the densely populated area of the city which are considered and operated by the Isfahan Department of Environment. In all of these stations, the real-time beta attenuation continuous mass monitor system was used to measure the PM_2.5_ concentrations.

The daily meteorological data corresponding to the 7 stations was collected from the Isfahan Meteorological Administration. Meteorological factors such as maximum, minimum and average daily air temperature, relative humidity, total rainfall and /or snowmelt, wind direction, and average and maximum sustained wind speed, are utilized in the calculation and screening processes.

The information regarding the collected data can be found in Table 1. Data from January 2011 to December 2019 was collected and analyzed, amounting to a total of 3285 days, which is equivalent to 9 years. The 24-h average of PM_2.5_ was calculated through modelling, utilizing daily meteorological data.

**Table 1:** Information regarding the air pollutant and meteorological parameters utilized in this study along with collinearity statistics.

Abbreviation	Variable	Unit	Temporal frequency	Collinearity statistics	
				VIF(Before)	VIF(After)
PM_2.5_	Fine particulate matter with a diameter of 2.5 microns or less	µg/m^3^	Hourly	Dependent Variable	Dependent Variable
T	Average Temperature	°C	24 h Average	1288.58	4.49
T_max_	Maximum temperature	°C	Maximum daily	521.17	Removed
T_min_	Minimum temperature	°C	Minimum daily	401.72	Removed
RH	Average relative humidity	%	24 h Average	26.01	6.62
PP	Total rainfall and/or snowmelt	mm	24 h Average	2.14	2.02
WS	Average wind speed	Km/h	24 h Average	9.71	2.12
WM	Maximum sustained wind speed	Km/h	Maximum daily	6.82	Removed
WD	Wind direction	–	Maximum daily	2.1	1.99

To ensure accurate modelling results, it is important to address the potential collinearity among the input variables mentioned above. Collinearity occurs when there is a high correlation between two or more variables, which can adversely affect the model's performance. To identify and mitigate this issue, the data was inputted into SPSS software, and collinearity statistics analysis was conducted. The Variance Inflation Factor (VIF) was then calculated to assess the extent of collinearity. Higher VIF values indicate stronger collinearity between a variable and the others. Typically, values exceeding 10 are indicative of moderate to high collinearity. Consequently, based on these findings (Table 1), the Tmax, Tmin, and WM variables were excluded from the model's input to prevent collinearity-related complications from impacting the model's output.

Based on the EPA classification of AQI and PM_2.5_, as well as the range of recorded PM_2.5_ (11.05–98.10 µg/m^3^) during the study period^15^, PM_2.5_ concentrations were categorized into 4 grades as follows:

1. Good: 0–12 µg/m^3^ (AQI: 0–50)
2. Moderate: 12.1–35.4 µg/m^3^ (AQI: 51–100)
3. Unhealthy for sensitive groups: 35.5–55.4 µg/m^3^ (AQI: 101–150)
4. Unhealthy for all people: 55.5–150.4 µg/m^3^ (AQI: 151–200)

According to the received data, wind speed was recorded in 8 directions, including North, South, East, West, North East, North West, South East and South West.

### Modelling techniques

In this study, four artificial intelligence models were compared to identify the optimal model for predicting the concentration ranges of PM_2.5_, based on the metrological conditions. These models were Artificial Neural Network (ANN), Ensembles of Classification Trees Random Forest (RF), Support Vector Machine (SVM) and k-nearest Neighbor Classification (KNN).

#### SVM

The Support Vector Machine (SVM) is a supervised machine learning algorithm that can be employed for classification problems. The SVM algorithm treats each data item as a data point in an n-dimensional space, where n represents the number of features. Through the process of receiving labelled training data and conducting supervised training, the SVM algorithm constructs a hyperplane which is then utilized to classify new instances. The vectors or data points that are nearest to the hyperplane and influence its position are referred to as support vectors. SVM always constructs a hyperplane with a maximum margin, representing the greatest separation between data points^16^. The effectiveness of SVMs for classification greatly relies on their parameter settings, particularly the penalty factor C and the kernel parameter σ2. These parameters play a crucial role in determining the classification outcome. Typically, fine-tuning these parameters is based on empirical knowledge and experience^3^.

#### RF

A decision tree is a straightforward model used to classify examples, and learning it is considered one of the most effective approaches for supervised classification learning. Ensemble methods are utilized to enhance predictive performance by combining multiple decision trees. The fundamental concept behind ensemble models is that a collection of weaker learners can collectively create a stronger learner. Bagging and boosting are commonly employed techniques for implementing ensemble decision trees^17^.

#### KNN

The k-nearest neighbours (KNN) algorithm is an uncomplicated and practical supervised machine learning technique that is capable of addressing both classification and regression predictive tasks. However, the k-nearest neighbours (KNN) algorithm is primarily utilized in pattern recognition and regression problems. The underlying principle of KNN is based on the assumption that similar data points tend to be close to each other in the feature space. To determine the appropriate value of K, which represents the number of neighbouring data points, the algorithm is executed multiple times with varying values of K. The aim is to find the value of K that minimizes the number of errors encountered, while still ensuring the algorithm's capability to accurately predict outcomes when presented with new, unseen data^18^.

#### ANN

The concept of an artificial neural network involves programming computers to mimic the interconnected behaviour of brain cells. Here, the multilayer perceptron (MLP) was applied. An MLP is a class of feedforward artificial neural networks. The MLP architecture is composed of three layers. The input layer accepts inputs in various formats given by the programmer. The hidden layer lies between the input and output layers. It carries out calculations to identify concealed features and patterns. The output layer transforms the input through the hidden layer, ultimately producing the final output conveyed by this layer^19^.

### Modelling for PM_2.5_ prediction

The objective of this study was to assess the PM_2.5_ pollution level. While the RF, SVM, and KNN models directly address this task through modelling, the ANN model takes a different approach. It first predicts the concentration of PM_2.5_ and subsequently determines the corresponding pollution level based on the criteria outlined in section "Monitoring locations". The implementation of ANN, RF, SVM, and KNN models utilized MATLAB R2020b software with version 9.9.0.1467703. For the models developed in this study, the input data included the following parameters: T, Tmax, Tmin, RH, PP, WS, VM, WD, Day (Day of the month is a number between 1 and 30), month (a number between 1 and 12), Year (between 2011 and 2019), station (between 1 and 7). In SVM, RF and KNN models, PM_2.5_ grades were introduced to the model as an output variable. In the ANN model, the concentration of PM_2.5_ was given to the model as a continuous numerical output variable.

The ANN model in Matlab software was constructed using the Neural Net Toolbox (nntool). To forecast PM_2.5_ concentrations, a multilayer feed-forward backpropagation network was employed. The hidden layer utilized a tangent sigmoid activation function, while the output layer utilized a pure linear activation function. It should be noted that the number of neurons in the hidden layer greatly affects the performance of the ANN. The equations from previous studies were employed to calculate the ideal quantity of neurons in the hidden layer^20,21^. Afterwards, a total of 2299 datasets (70%) were randomly selected for training the ANN model, while 493 datasets (15%) were used for validation, and another 493 records (15%) were reserved for testing. The Levenberg–Marquardt backpropagation training algorithm (trainlm) was employed to train the ANN. After designing the optimal ANN architecture, the model was utilized to estimate the concentration of PM_2.5_ in the test dataset. The output predicted by the model was ranked according to the grades defined in this study so that the performance of the ANN model is comparable to the classification models.

The MATLAB software's classification learner app is designed to train models for data classification. This app includes various high-performing classification model types such as nearest neighbours, discriminant analysis, decision trees, logistic regression, support vector machines, naive Bayes, ensemble classification, and kernel approximation^22^. After conducting a preliminary evaluation, three classification algorithms namely SVM, RF, and KNN were chosen from various algorithms available in the toolbox. The performance of these classifiers was assessed for predicting PM_2.5_ grades in this research.

The optimal SVM model structure was determined by optimizing kernel function, box constraint level, kernel scale, and multiclass method parameters. Also, to determine the best KNN model, the number of neighbours, distance metric and distance weight parameters were optimized. The parameters of the RF model, such as the ensemble method, maximum number of splits, number of learners, and learning rate, were optimized. Parameter tuning was conducted by stepwise optimization.

To prevent overfitting during the implementation of SVM, KNN, and RF models, a holdout validation is conducted. In this process, 15% of the original dataset is set aside as a test dataset. Then, the holdout validation method is applied, where 15% of the dataset is allocated for validation and the remaining portion is used for training. This helps ensure the models' performance is not biased by the training data. Holdout Validation divides the input data into the training set and the validation set of the obtained model. These two sets are complementary. Holdout Validation is utilized for big datasets^23^. The test dataset was used to extract and evaluate the trained classifier models.

Confusion matrix, also known as an error matrix, and cross-entropy loss, also referred to as log loss, are used in machine learning to evaluate the effectiveness of classification models. In this particular study, both of these techniques were utilized to assess the performance of an ANN for classification purposes. A confusion matrix is a summary table that shows how well the model has performed in predicting samples of different classes. When optimizing classification models and generally calculating the difference between the predicted and actual responses, cross-entropy can be used as a loss function^20^. After analyzing the confusion matrix, various performance parameters, including sensitivity, specificity, F1 score, accuracy, precision, and the area under the curve (AUC), are calculated to assess the models' performance.

Accuracy is defined as the proportion of correct predictions out of all the predictions made. Sensitivity, also known as the true positive rate, is calculated as the proportion of correctly predicted positive instances to the total number of actual positive instances. Specificity, also known as the true negative rate, refers to the proportion of accurately predicted negative instances in relation to the total number of actual negative instances. Precision, also known as positive predictive value, refers to the proportion of correctly predicted positive instances compared to the total number of instances that were predicted as positive. The F1 score can be defined as the harmonic mean of sensitivity and precision, two important parameters in data analysis. The receiver operating characteristic curve (ROC) is a graphical representation of the probability of a model's performance. The area under the curve (AUC) quantifies the separability or discriminative power of the model in distinguishing between different classes^24^. Equations (1–5) can be utilized to compute these parameters^25^.

1 $$Sensitivity=\frac{TP}{TP+FN}$$

2 $$Specificity=\frac{TN}{FP+TN}$$

3 $$Precision=\frac{TP}{TP+FP}$$

4 $$Accuracy=\frac{TP+TN}{TP+TN+FP+FN}$$

5 $$F1 Score=2*\frac{Sensitivity* Precision}{Sensitivity + Precision}$$

Here, TP, TN, FP, and FN are True Positive, True Negative, False Positive, and False Negative, respectively^20,26^.

The parameters used to evaluate the performance of ANN were the correlation coefficient (R) and the mean squared error (MSE), as shown in Eqs. (6) and (7)^27,28^.

6 $$R=\sqrt{\frac{1-\sum_{i=1}^{n}{({Y}_{p}-{Y}_{e})}^{2}}{\sum_{i=1}^{n}{({Y}_{p}-{\overline{Y} }_{e})}^{2}} }$$

7 $$MSE=\frac{1}{n}\sum_{i=1}^{n}{({Y}_{e}-{Y}_{p})}^{2}$$

where n is the number of experimental data, and ${Y}_{p}$, ${Y}_{e}$, and ${\overline{Y} }_{e}$ represent the predicted value, the experimental value, and the average of experimental data for the output variable, respectively.

As mentioned earlier, the output predicted by the model was graded according to the stratification defined in this study, so that the performance of the ANN model becomes comparable with the classification models. Afterwards, the confusion matrix of the neural network was also determined. After determining the best model, for external validation, meteorological data for 2020 was received and introduced to the optimal model, and PM_2.5_ was predicted for monitoring stations for each month in 2020. Finally, by entering the data of 2020 into the ArcGIS 10.8 Version 10.7.0.10450 software, the pollution map was created for each month of the year.

## Results and discussion

### Evaluation of the collected data

**Figure 2:**
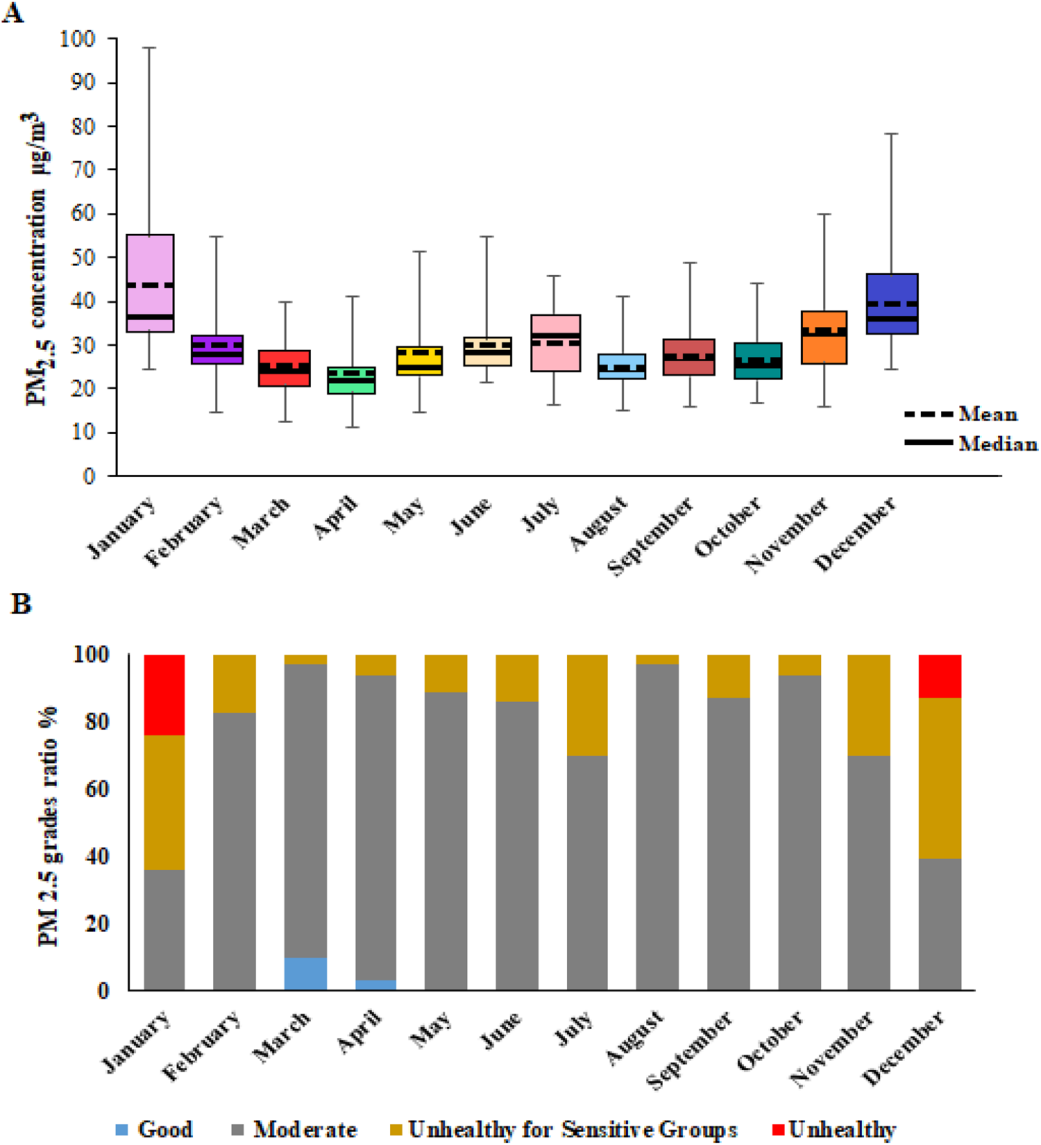
(**A**) PM_2.5_ concentration, and (**B**) Probability of different PM_2.5_ grades in all stations.

Figure 2A shows a box plot for the concentration of PM_2.5_ in different months of the year. In this graph, the values measured in all 7 stations over 9 years can be seen separately for each month. According to this graph, January and December are the most polluted months of the year, as well as March and April have the best air quality with the lowest concentration of PM_2.5_.

**Figure 3:**
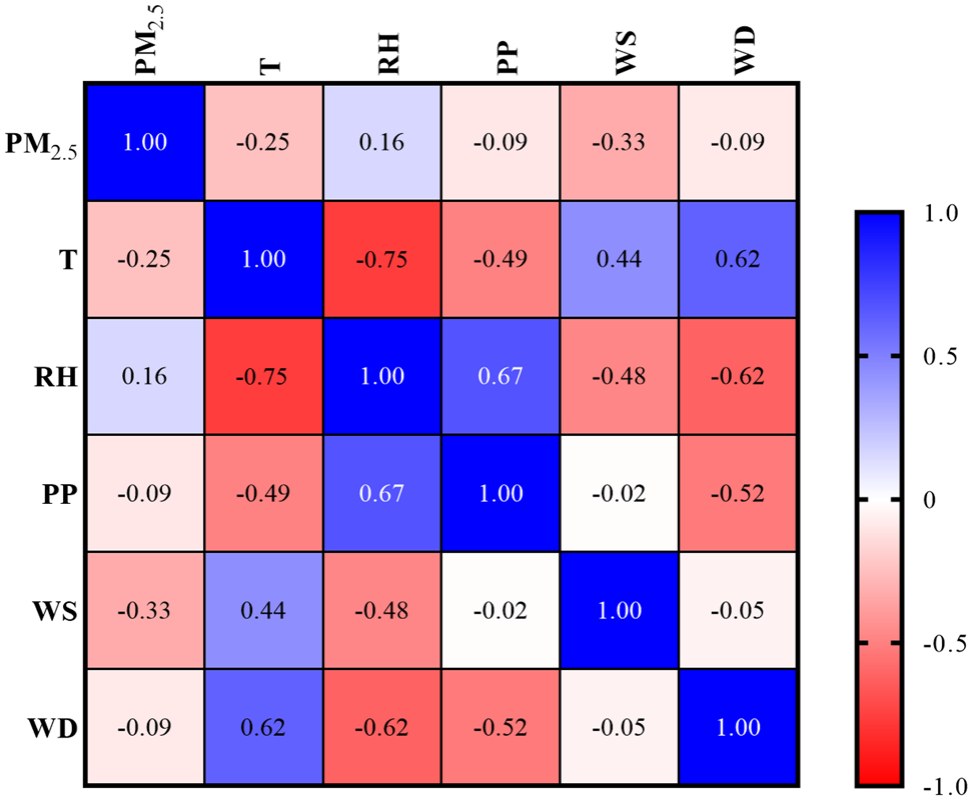
Correlation heat map between variables (The abbreviation of the variables is defined in Table 1).

To draw Fig. 2B, firstly, based on the concentration, the PM_2.5_ grade is specified for each day, and then based on the number of repetitions of each grade per month during 9 years, this graph was drawn. RStudio version 1.3.959 software and the following packages were used to draw Fig. 3.

Package ggplot2 (https://cran.r-project.org/web/packages/ggplot2/index.html) and package correlation (https://cran.r-project.org/web/packages/correlation/index.html).

Spearman correlation was performed on the data at first. Then a heat map was created to display the correlation results graphically (Fig. 3). A heat map shows the value of the correlation coefficient between each possible pair of variables. Based on this diagram, all the selected variables have a high correlation with PM_2.5_ and are suitable for modelling.

### Results classification

To determine the most suitable model for predicting the amount of pollution caused by PM_2.5_, the best structure of SVM, KNN, RF and ANN models were determined at first.

**Figure 4:**
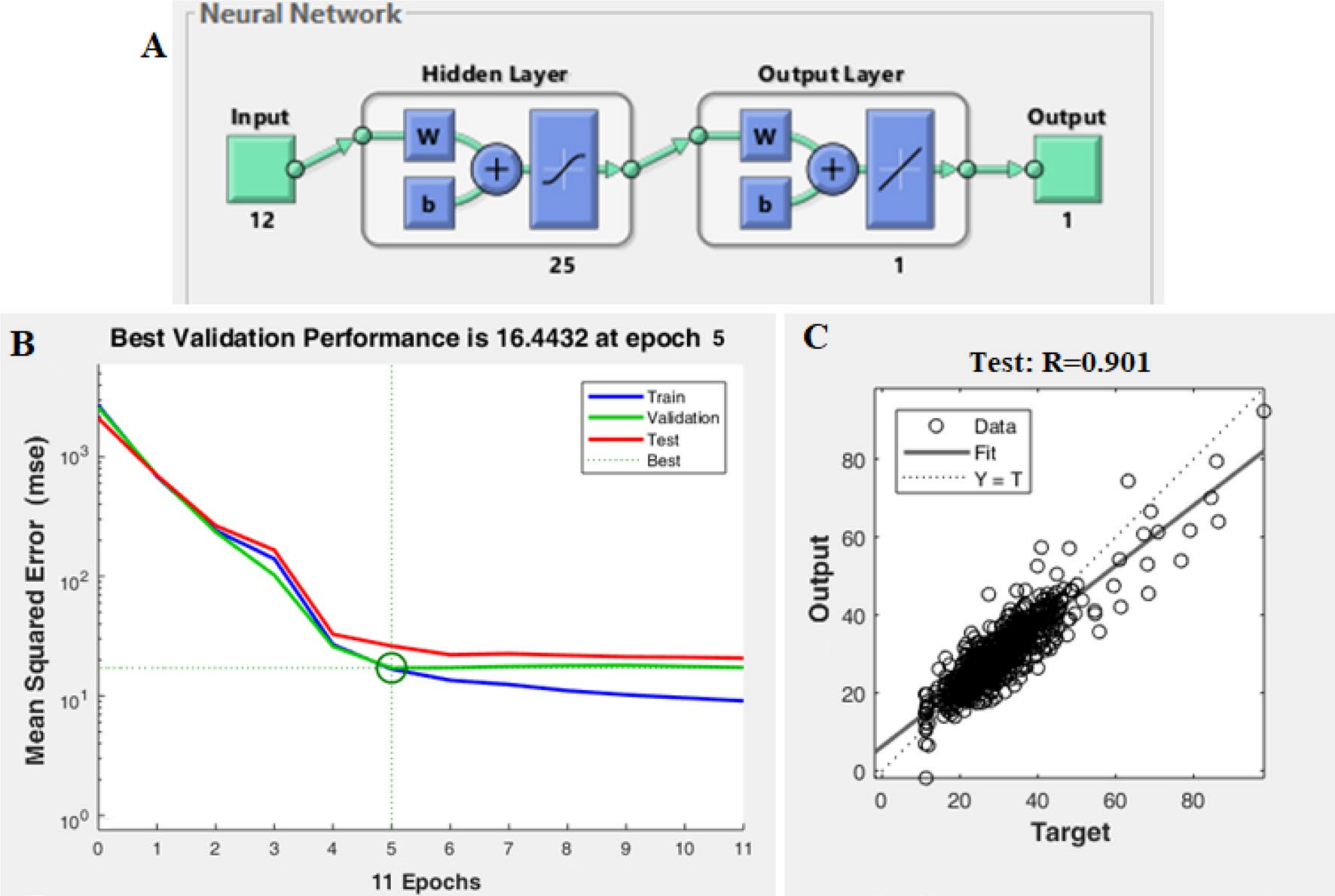
(**A**) The ANN structure, (**B**) The MSE error graph in the output of ANN, (**C**) The regression plot of the test dataset.

The highest performance of the ANN was achieved using 25 neurons in the hidden layer. The number of neurons for the hidden layer was determined within a range of 6 to 230, based on calculations derived from published equations^21^. The final ANN model structure (9:25:1) is shown in Fig. 4A.

The graph in Fig. 4B illustrates the relationship between the number of epochs (iterations) and the errors for the training, validation, and testing phases of the ANN model. Typically, the error decreases after several training epochs, and the training algorithm stops if the validation error increases for six consecutive iterations or if the maximum error per epoch is exceeded. According to Fig. 6B, the training algorithms are stopped after 11 epochs due to the validation error, even though the training errors are sufficiently low. The test and validation error plots exhibit comparable patterns, suggesting that the data in this study has been appropriately divided.

The regression plot shown in Fig. 4C illustrates the network outputs compared to the targets of the test dataset. Ideally, the data points should align along a 45-degree line, indicating a perfect fit where the network outputs and targets are equal. Upon observation, it is evident that the output closely matches the targets during testing. This study concludes that the network response is satisfactory, and the simulation can be utilized for inputting new data.

As mentioned earlier, the output predicted by the model was graded according to the stratification defined in this study so that the performance of the ANN model be comparable with the classification models. Afterwards, the confusion matrix of the neural network was also determined.

To determine the optimal internal structure of each model, various internal parameters were set to assess the accuracy level. The model with the highest accuracy was identified as the best-fit model. Owing to the number of iterations performed to achieve the best model, the results of each step involved are not provided. However, Table 2 showcases the results obtained from the best-fit model.

**Table 2:** Performance parameters of ANN, Quadratic SVM and RF for test data.

Model	Accuracy	Sensitivity	Specificity	Precision	F1 Score
ANN	0.911	0.889	0.720	0.887	0.882
SVM	0.848	0.833	0.529	0.823	0.813
KNN	0.823	0.807	0.424	0.793	0.772
RF	0.861	0.848	0.615	0.842	0.835

In the evaluation of kernel functions in the SVM model, various options including linear, quadratic, cubic, fine Gaussian, medium Gaussian, and coarse Gaussian were investigated and the accuracy of each was equal to 82.4%, 84.8%, 83.5%, 80.7% and 81.7% respectively. Based on the accuracy level, the quadratic function was determined to be the most suitable choice. Also, the SVM model showed the best performance when kernel scale, box constraint level and multiclass method are set to 1, 1 and one versus one.

In the KNN model, for distance metric options the Fine, Medium, Coarse, Cosine, Cubic and Weighted KNN were evaluated and the accuracy of each was equal to 78.9%, 76.3%, 74.3%, 82.3%, 76.1% and 81.9% respectively. So based on the accuracy level, the Cosine function was selected as the best one. Also, the KNN model showed the best performance when the number of neighbours and distance weight were set to 10 and equal.

Also, for the RF model, Ensemble methods were applied, which included Bagging and Boosting techniques. The Boosting technique was selected as the best one based on the accuracy level (Bagging 84.4%, Boosting 86.1%). Also, the RF model showed the best performance when the maximum number of splits, number of learners and learning rate are set to 20, 30 and 0.1.

**Figure 5:**
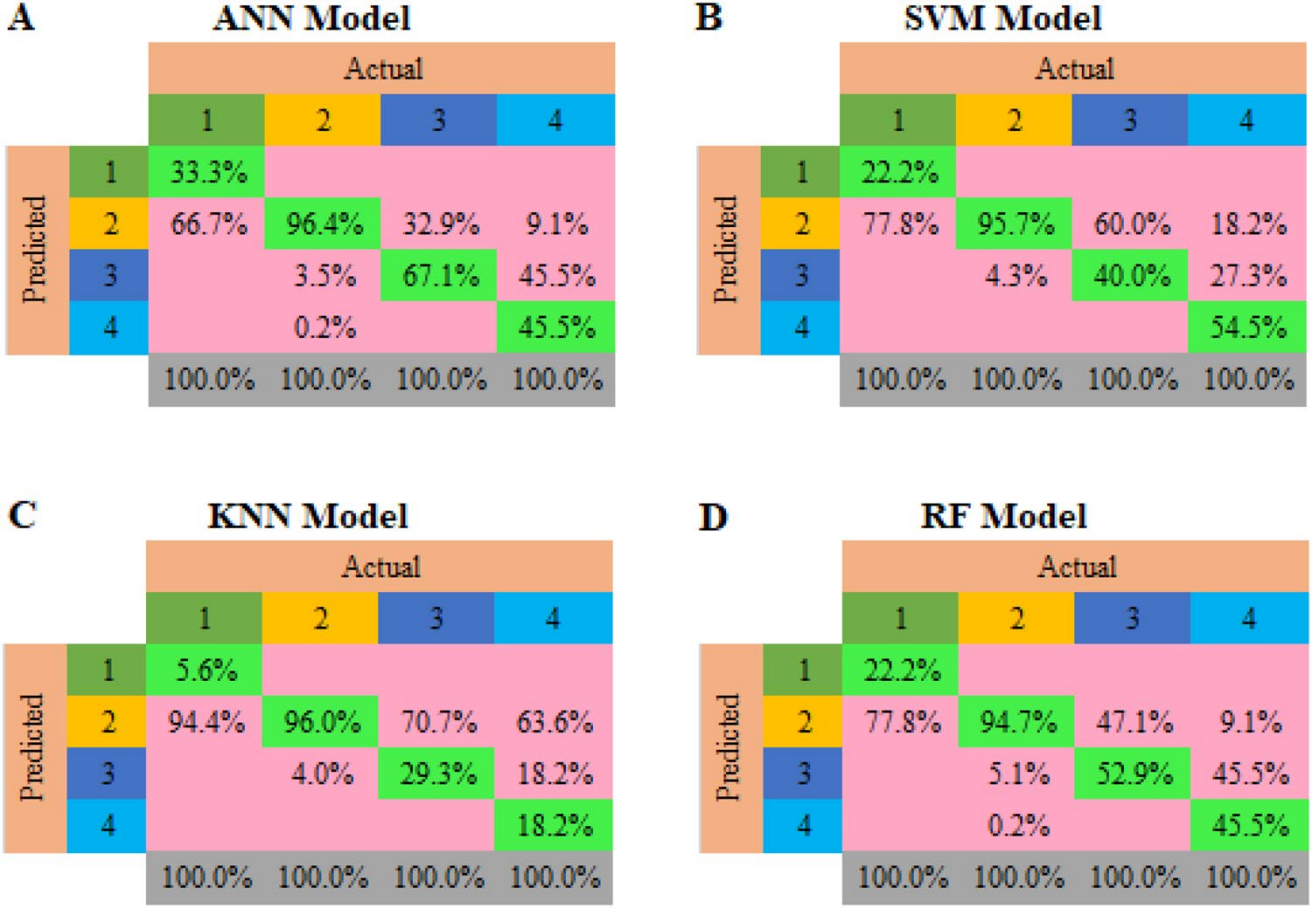
Confusion matrix of (**A**) ANN, (**B**) SVM, (**C**) KNN and (**D**) RF models.

As previously stated in this study, Fig. 5 displays the confusion matrices that were utilized to examine the performance of ANN, SVM, KNN, and RF classification models in predicting PM_2.5_ grades.

In addition, Table 2 compares the accuracy, sensitivity, specificity, precision, recall, and F1 scores of different models. The ANN algorithm achieved the highest accuracy (90.1%), followed by RF, SVM, and KNN algorithms, respectively. In all algorithms, Sensitivity was higher than Specificity, but again, the ANN showed the best results, followed by the RF. In terms of Precision and F1 Score parameters, ANN and RF models are in the first and second ranks.

**Figure 6:**
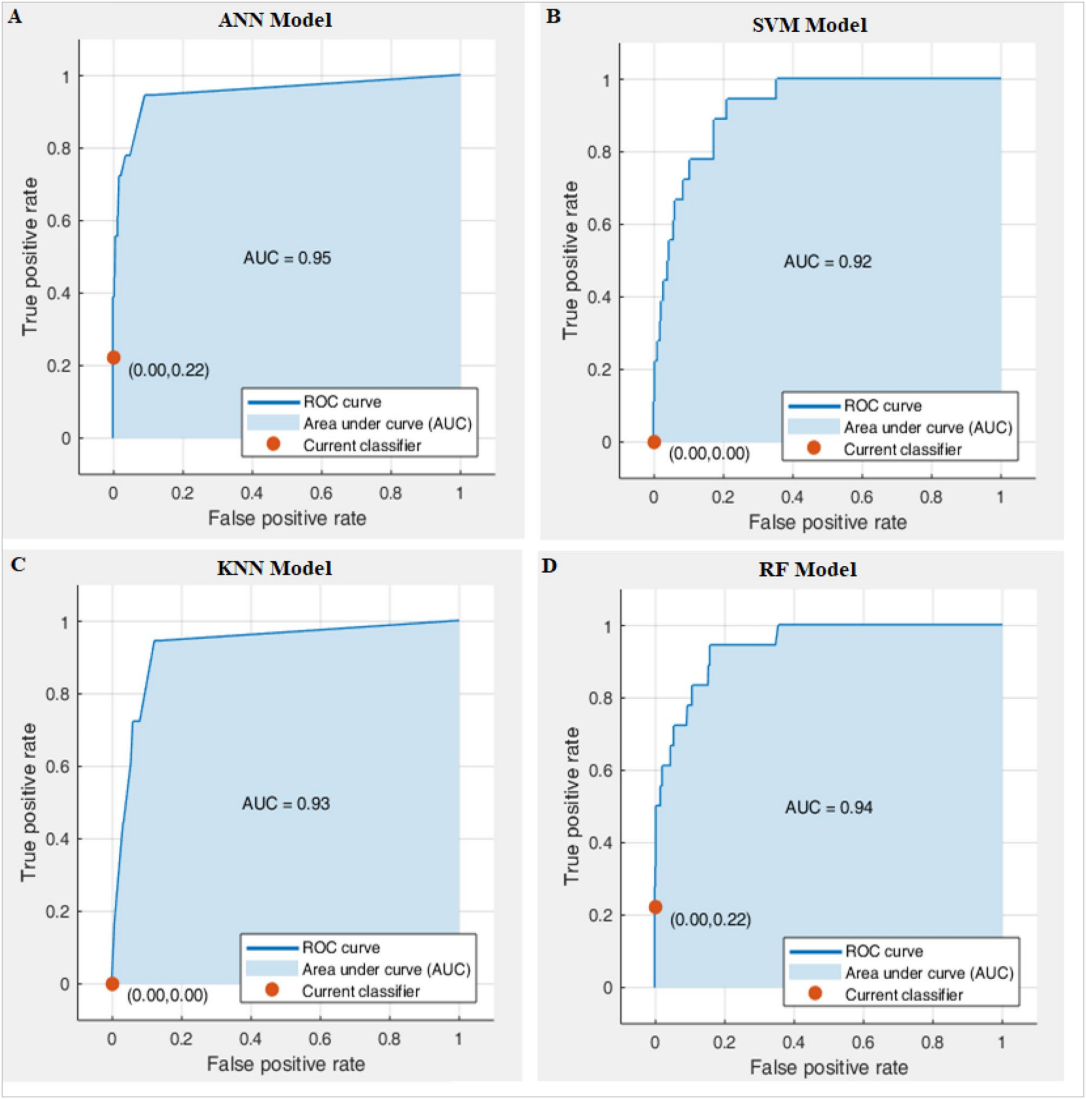
ROC curves of (**A**) ANN, (**B**) SVM, (**C**) KNN and (**D**) RF Models.

Figure 6 also shows the ROC curves and the area under them. The AUC of the artificial neural network model was 0.95, which outperformed the AUC of the random forest model (0.94), k-nearest neighbours model (0.93), and support vector machine model (0.92).

Therefore, based on the obtained results, the performance of the artificial neural network in predicting the grade of pollution caused by PM_2.5_ significantly is more suitable than the others, followed by the random forest as a classifier algorithm based on the meteorological parameters.

In 2020, Xu et al. created a sophisticated prediction model for accurately estimating the 24-h concentration levels of station-specific and regional PM_2.5_. This model consists of two components: a site prediction model called the TSRT model, which is a regression model based on a regression-tree algorithm, and a grid prediction model known as the ANN model, which utilizes deep learning techniques. The method was assessed using data gathered from monitoring stations located in the Beijing-Tianjin-Hebei region. They used meteorological data, highway network data, and air quality data. The findings suggest that the TSRT model outperforms the grid prediction model with an average R^2^ of 0.9 and an average MAE value of 10.35 μg/m^3^^6^.

Liu et al. in 2019, to predict the PM_2.5_ grades using meteorological pattern data, Compared SVM and ANN models. They used the particle swarm optimization (PSO) algorithm to improve SVM performance. In some parameters, the PSO-SVM model has better performance and in others, the ANN model was more appropriate. For example, the accuracy, precision, recall, F1-score and AUC for PSO-SVM and ANN respectively obtained, (73.52% and 73.17%), (0.91 and 0.78), (0.75 and 0.89), (0.82 and 0.83) and (0.82 and 0.75)^3^.

In 2021, Sayeed et al. employed a generalized deep convolutional neural network (CNN) model to predict air pollutants, including PM_10_, PM_2.5_, and NO_2_, for seven days^29^. In a study conducted by Zhang et al. in 2021, they utilized a random forest model to assess the levels of ambient PM_2.5_ in the industrialized area of South Africa. The model incorporated various inputs such as satellite aerosol optical depth (AOD), meteorological data, land use information, and socioeconomic data. Through cross-validation, the model achieved an R^2^ value of 0.80 and a root mean square error of 9.40 μg/m^3^^30^.

In 2020, Lin et al. created a model using RF and XGBoost techniques to forecast the levels of PM_2.5_ and nitrate concentrations at a road site station. The model successfully achieved a strong correlation between the predicted and observed PM_2.5_ values, with an R^2^ value of 0.81. The root mean square error (RMSE) and mean absolute error (MAE) were measured at 6.81 μg/m^3^ and 5.10 μg/m^3^ respectively, indicating a reasonable level of accuracy in the predictions^31^.

In 2021, Bera et al. conducted a study to predict PM_2.5_ concentrations in Kolkata Metropolitan City, India. They utilized multiple linear regression and artificial neural network models and compared their accuracy levels. PM_2.5_ data was obtained from the State Pollution Control Board and daily meteorological data were collected from the World Weather website. The findings indicate that the ANN model outperformed the MLR model. During the testing phase, the ANN model demonstrated an RMSE of 2.55, MAE of 4.32, and R^2^ value of 0.69.^32^.

### External validation and GIS implementation

**Figure 7:**
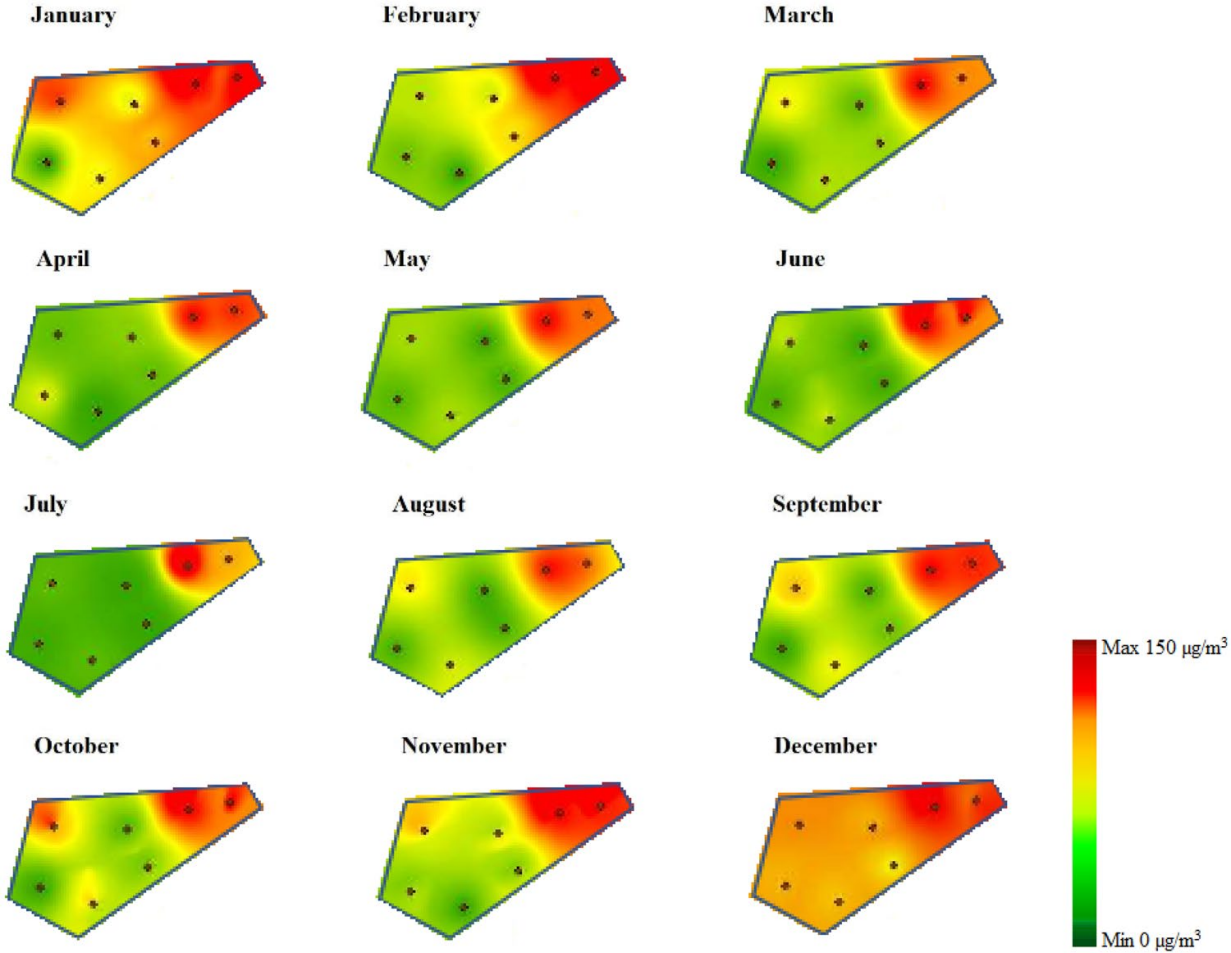
The output of the ANN model implemented in Arc GIS software for PM_2.5_ pollution in 2020.

Considering that the ANN model showed the best performance in predicting the amount of PM_2.5_, the meteorological and PM_2.5_ data of the monitoring sites in 2020 were used for external validation. The data related to the monthly average of each station was introduced to the optimal neural network model and the concentration of PM_2.5_ in each station was calculated. The amount of R and MSE for external validation data was achieved at 0.895 and 14.382, respectively. Finally, by entering the predicted data for each station in 2020 into the ArcGIS 10.8 software and using the IDW (Inverse Distance Weighting) method, interpolation was conducted on the area of Isfahan city, and the pollution map was drawn for each month of the year (Fig. 7). The central area of Isfahan City, where the PM_2.5_ monitoring stations are located and the populous area of the city, was chosen to draw the pollution map. IDW interpolation is a popular technique used in GIS software for spatial data analysis. It is designed to estimate unknown values at specific locations based on the known values from surrounding data points. The IDW method uses various techniques for interpolation, such as polynomial or spline interpolation. These techniques work by fitting a mathematical curve or function to the known data points and then estimating values along that curve^33^. As Fig. 7 shows, in cold months e.g. December and January, the atmospheric concentration of PM_2.5_ is much higher than in other months due to the occurrence of a stable atmosphere and thermal inversion phenomenon. Within the city areas, the northeast part of the city is more polluted compared to the other areas, because of its densely populated and high traffic condition. Isfahan is one of the most populated cities with more than 2 million residents and a main industrial hub in Iran. On one hand, emissions from different industries such as iron ore, steel, cement, oil refinery and petrochemical industries, and two power plants located around the city as well as emissions from the daily traffic of nearly 2 million motor vehicles in the city under the stable weather condition, and in the other hand mismanagement and lack of an integrated air pollution reduction schemes has increased air pollution levels in Isfahan.

The main advantage of this study is that 4 artificial intelligence models were compared in predicting the amount of PM_2.5_ using meteorological data, which has not been compared in other studies. Additionally, when comparing it to widely recognized interpolation models like kriging interpolation, the technique developed in this study shows the ability to accurately predict PM_2.5_ concentrations for each day throughout the year. As a result, this approach has the potential to enhance PM_2.5_ predictions, ultimately benefiting individuals' daily lives and having an impact on policy-making decisions.

## Conclusion

In this study, to determine the best model for the prediction of atmospheric PM_2.5_ levels based on weather conditions in Isfahan City, a populous and industrialized city of central Iran, four artificial intelligence models were applied and their accuracy was compared. These models include Artificial Neural Networks (ANN), ensembles of classification trees random forest (RF) and Support Vector Machine (SVM) as well as k-nearest neighbour classification (KNN). The daily meteorological data and PM_2.5_ concentrations of the 7 monitoring stations in Isfahan City were collected in 3285 days (2011–2019). Meteorological factors, including maximum, minimum and average daily air temperature, relative humidity, total rainfall and/or snowmelt, average wind speed, maximum sustained wind speed and wind direction, are used for calculation and screening. The performance of classification models in machine learning was analyzed using a confusion matrix and cross-entropy loss. Various parameters, including accuracy, sensitivity, specificity, precision, F1 score, and the area under the curve (AUC), were calculated based on the obtained confusion matrix to evaluate the models' performance. The ANN algorithm achieved the highest accuracy of 91.1%, followed by RF, SVM, and KNN algorithms in consecutive rankings. In all algorithms, Sensitivity was higher than Specificity, but again, the ANN showed the best results, followed by the RF. In terms of Precision and F1 Score parameters, ANN and RF models were in the first and second ranks. The AUC of the ANN model (0.95) was greater than the AUC of the RF (0.94), KNN (0.93) and SVM models (0.92).

Therefore, according to the results, the artificial neural network was the most suitable model for the prediction of the pollution grade of PM_2.5_, followed by the random forest as a classifier algorithm based on the meteorological conditions. The final ANN model structure was 9:25:1. Tangent sigmoid, and pure line activation functions were applied in the hidden and output layers. Levenberg–Marquardt backpropagation training algorithm (trainlm) was used to train ANN.

Eventually, by introducing the predicted data for each station in 2020 into the ArcGIS software and using the IDW method, interpolation was done on the area of Isfahan City and the pollution map was drawn in each month of the year.

### Supplementary Information

Supplementary Table S1.

## Data Availability

The supporting data are available from the corresponding authors upon reasonable request.
