# Supplementary material for: Prediction of atmospheric PM2.5 level by machine learning techniques in Isfahan, Iran
Source: Sci Rep. 2024 Jan 24;14:2109. doi: 10.1038/s41598-024-52617-z (PMC10808097; doi:10.1038/s41598-024-52617-z)
Supplement: Supplementary file 1 — Supplementary Table S1. [file 41598_2024_52617_MOESM1_ESM.docx]

**Supplementary file**

Table S1: 2020 Data used for external validation

| year | month | | Day | site | T | Tmax | Tmin | RH | PP | V | WS | VM | WD | PM 2.5 actual |
| --- | --- | --- | --- | --- | --- | --- | --- | --- | --- | --- | --- | --- | --- | --- |
| 2020 | January | 1 | 15 | 1 | 1.6 | 10 | -7 | 53.7 | 6.08 | 8.5 | 9.3 | 20.7 | 1 | 41.55584 |
| 2020 | January | 1 | 15 | 2 | 1.6 | 10 | -7 | 53.7 | 6.08 | 8.5 | 9.3 | 20.7 | 1 | 33.92209 |
| 2020 | January | 1 | 15 | 3 | 1.6 | 10 | -7 | 53.7 | 6.08 | 8.5 | 9.3 | 20.7 | 1 | 35.06503 |
| 2020 | January | 1 | 15 | 4 | 1.6 | 10 | -7 | 53.7 | 6.08 | 8.5 | 9.3 | 20.7 | 1 | 36.01602 |
| 2020 | January | 1 | 15 | 5 | 1.6 | 10 | -7 | 53.7 | 6.08 | 8.5 | 9.3 | 20.7 | 1 | 35.43903 |
| 2020 | January | 1 | 15 | 6 | 1.6 | 10 | -7 | 53.7 | 6.08 | 8.5 | 9.3 | 20.7 | 1 | 34.33475 |
| 2020 | January | 1 | 15 | 7 | 1.6 | 10 | -7 | 53.7 | 6.08 | 8.5 | 9.3 | 20.7 | 1 | 33.71055 |
| 2020 | February | 2 | 15 | 1 | 6.1 | 15.9 | -4 | 35.3 | 13.46 | 8.9 | 11.8 | 27.6 | 1 | 37.05586 |
| 2020 | February | 2 | 15 | 2 | 6.1 | 15.9 | -4 | 35.3 | 13.46 | 8.9 | 11.8 | 27.6 | 1 | 29.72829 |
| 2020 | February | 2 | 15 | 3 | 6.1 | 15.9 | -4 | 35.3 | 13.46 | 8.9 | 11.8 | 27.6 | 1 | 30.03556 |
| 2020 | February | 2 | 15 | 4 | 6.1 | 15.9 | -4 | 35.3 | 13.46 | 8.9 | 11.8 | 27.6 | 1 | 30.48771 |
| 2020 | February | 2 | 15 | 5 | 6.1 | 15.9 | -4 | 35.3 | 13.46 | 8.9 | 11.8 | 27.6 | 1 | 29.63456 |
| 2020 | February | 2 | 15 | 6 | 6.1 | 15.9 | -4 | 35.3 | 13.46 | 8.9 | 11.8 | 27.6 | 1 | 28.26125 |
| 2020 | February | 2 | 15 | 7 | 6.1 | 15.9 | -4 | 35.3 | 13.46 | 8.9 | 11.8 | 27.6 | 1 | 27.47711 |
| 2020 | March | 3 | 15 | 1 | 9.5 | 18.1 | 0.4 | 40.3 | 20.83 | 8.9 | 12.2 | 32.2 | 1 | 37.33333 |
| 2020 | March | 3 | 15 | 2 | 9.5 | 18.1 | 0.4 | 40.3 | 20.83 | 8.9 | 12.2 | 32.2 | 1 | 30.30162 |
| 2020 | March | 3 | 15 | 3 | 9.5 | 18.1 | 0.4 | 40.3 | 20.83 | 8.9 | 12.2 | 32.2 | 1 | 30.02276 |
| 2020 | March | 3 | 15 | 4 | 9.5 | 18.1 | 0.4 | 40.3 | 20.83 | 8.9 | 12.2 | 32.2 | 1 | 29.56275 |
| 2020 | March | 3 | 15 | 5 | 9.5 | 18.1 | 0.4 | 40.3 | 20.83 | 8.9 | 12.2 | 32.2 | 1 | 28.07139 |
| 2020 | March | 3 | 15 | 6 | 9.5 | 18.1 | 0.4 | 40.3 | 20.83 | 8.9 | 12.2 | 32.2 | 1 | 26.73202 |
| 2020 | March | 3 | 15 | 7 | 9.5 | 18.1 | 0.4 | 40.3 | 20.83 | 8.9 | 12.2 | 32.2 | 1 | 26.3861 |
| 2020 | April | 4 | 15 | 1 | 14.7 | 22.3 | 6.4 | 43.2 | 32.52 | 8.5 | 13 | 35.4 | 1 | 39.31694 |
| 2020 | April | 4 | 15 | 2 | 14.7 | 22.3 | 6.4 | 43.2 | 32.52 | 8.5 | 13 | 35.4 | 1 | 32.23838 |
| 2020 | April | 4 | 15 | 3 | 14.7 | 22.3 | 6.4 | 43.2 | 32.52 | 8.5 | 13 | 35.4 | 1 | 33.8365 |
| 2020 | April | 4 | 15 | 4 | 14.7 | 22.3 | 6.4 | 43.2 | 32.52 | 8.5 | 13 | 35.4 | 1 | 35.82879 |
| 2020 | April | 4 | 15 | 5 | 14.7 | 22.3 | 6.4 | 43.2 | 32.52 | 8.5 | 13 | 35.4 | 1 | 37.06062 |
| 2020 | April | 4 | 15 | 6 | 14.7 | 22.3 | 6.4 | 43.2 | 32.52 | 8.5 | 13 | 35.4 | 1 | 38.88028 |
| 2020 | April | 4 | 15 | 7 | 14.7 | 22.3 | 6.4 | 43.2 | 32.52 | 8.5 | 13 | 35.4 | 1 | 41.88417 |
| 2020 | May | 5 | 15 | 1 | 22 | 30.9 | 11 | 24 | 10.16 | 8.7 | 12.6 | 34.8 | 1 | 33.50543 |
| 2020 | May | 5 | 15 | 2 | 22 | 30.9 | 11 | 24 | 10.16 | 8.7 | 12.6 | 34.8 | 1 | 25.43557 |
| 2020 | May | 5 | 15 | 3 | 22 | 30.9 | 11 | 24 | 10.16 | 8.7 | 12.6 | 34.8 | 1 | 23.63758 |
| 2020 | May | 5 | 15 | 4 | 22 | 30.9 | 11 | 24 | 10.16 | 8.7 | 12.6 | 34.8 | 1 | 21.37366 |
| 2020 | May | 5 | 15 | 5 | 22 | 30.9 | 11 | 24 | 10.16 | 8.7 | 12.6 | 34.8 | 1 | 18.61489 |
| 2020 | May | 5 | 15 | 6 | 22 | 30.9 | 11 | 24 | 10.16 | 8.7 | 12.6 | 34.8 | 1 | 17.15545 |
| 2020 | May | 5 | 15 | 7 | 22 | 30.9 | 11 | 24 | 10.16 | 8.7 | 12.6 | 34.8 | 1 | 17.84154 |
| 2020 | June | 6 | 15 | 1 | 27.6 | 37.1 | 15.9 | 14.2 | 0 | 9 | 12.9 | 30.8 | 3 | 53.9546 |
| 2020 | June | 6 | 15 | 2 | 27.6 | 37.1 | 15.9 | 14.2 | 0 | 9 | 12.9 | 30.8 | 3 | 40.52869 |
| 2020 | June | 6 | 15 | 3 | 27.6 | 37.1 | 15.9 | 14.2 | 0 | 9 | 12.9 | 30.8 | 3 | 37.45582 |
| 2020 | June | 6 | 15 | 4 | 27.6 | 37.1 | 15.9 | 14.2 | 0 | 9 | 12.9 | 30.8 | 3 | 36.44564 |
| 2020 | June | 6 | 15 | 5 | 27.6 | 37.1 | 15.9 | 14.2 | 0 | 9 | 12.9 | 30.8 | 3 | 36.11336 |
| 2020 | June | 6 | 15 | 6 | 27.6 | 37.1 | 15.9 | 14.2 | 0 | 9 | 12.9 | 30.8 | 3 | 37.08336 |
| 2020 | June | 6 | 15 | 7 | 27.6 | 37.1 | 15.9 | 14.2 | 0 | 9 | 12.9 | 30.8 | 3 | 39.48696 |
| 2020 | July | 7 | 15 | 1 | 29.4 | 37.9 | 18.7 | 16.9 | 0 | 9.1 | 11.2 | 31.7 | 5 | 47.1427 |
| 2020 | July | 7 | 15 | 2 | 29.4 | 37.9 | 18.7 | 16.9 | 0 | 9.1 | 11.2 | 31.7 | 5 | 39.79099 |
| 2020 | July | 7 | 15 | 3 | 29.4 | 37.9 | 18.7 | 16.9 | 0 | 9.1 | 11.2 | 31.7 | 5 | 37.86214 |
| 2020 | July | 7 | 15 | 4 | 29.4 | 37.9 | 18.7 | 16.9 | 0 | 9.1 | 11.2 | 31.7 | 5 | 36.94823 |
| 2020 | July | 7 | 15 | 5 | 29.4 | 37.9 | 18.7 | 16.9 | 0 | 9.1 | 11.2 | 31.7 | 5 | 36.82258 |
| 2020 | July | 7 | 15 | 6 | 29.4 | 37.9 | 18.7 | 16.9 | 0 | 9.1 | 11.2 | 31.7 | 5 | 38.08989 |
| 2020 | July | 7 | 15 | 7 | 29.4 | 37.9 | 18.7 | 16.9 | 0 | 9.1 | 11.2 | 31.7 | 5 | 40.98566 |
| 2020 | August | 8 | 15 | 1 | 28.8 | 37.8 | 17.6 | 18.6 | 0.51 | 9.2 | 10.7 | 26.7 | 3 | 59.5339 |
| 2020 | August | 8 | 15 | 2 | 28.8 | 37.8 | 17.6 | 18.6 | 0.51 | 9.2 | 10.7 | 26.7 | 3 | 43.17228 |
| 2020 | August | 8 | 15 | 3 | 28.8 | 37.8 | 17.6 | 18.6 | 0.51 | 9.2 | 10.7 | 26.7 | 3 | 37.23885 |
| 2020 | August | 8 | 15 | 4 | 28.8 | 37.8 | 17.6 | 18.6 | 0.51 | 9.2 | 10.7 | 26.7 | 3 | 33.58927 |
| 2020 | August | 8 | 15 | 5 | 28.8 | 37.8 | 17.6 | 18.6 | 0.51 | 9.2 | 10.7 | 26.7 | 3 | 32.22562 |
| 2020 | August | 8 | 15 | 6 | 28.8 | 37.8 | 17.6 | 18.6 | 0.51 | 9.2 | 10.7 | 26.7 | 3 | 33.17645 |
| 2020 | August | 8 | 15 | 7 | 28.8 | 37.8 | 17.6 | 18.6 | 0.51 | 9.2 | 10.7 | 26.7 | 3 | 35.56697 |
| 2020 | September | 9 | 15 | 1 | 22.6 | 32.5 | 10.9 | 19.3 | 0 | 9.3 | 8.5 | 21.2 | 1 | 57.44809 |
| 2020 | September | 9 | 15 | 2 | 22.6 | 32.5 | 10.9 | 19.3 | 0 | 9.3 | 8.5 | 21.2 | 1 | 40.14322 |
| 2020 | September | 9 | 15 | 3 | 22.6 | 32.5 | 10.9 | 19.3 | 0 | 9.3 | 8.5 | 21.2 | 1 | 34.62034 |
| 2020 | September | 9 | 15 | 4 | 22.6 | 32.5 | 10.9 | 19.3 | 0 | 9.3 | 8.5 | 21.2 | 1 | 30.90459 |
| 2020 | September | 9 | 15 | 5 | 22.6 | 32.5 | 10.9 | 19.3 | 0 | 9.3 | 8.5 | 21.2 | 1 | 28.14616 |
| 2020 | September | 9 | 15 | 6 | 22.6 | 32.5 | 10.9 | 19.3 | 0 | 9.3 | 8.5 | 21.2 | 1 | 26.54144 |
| 2020 | September | 9 | 15 | 7 | 22.6 | 32.5 | 10.9 | 19.3 | 0 | 9.3 | 8.5 | 21.2 | 1 | 25.36314 |
| 2020 | October | 10 | 15 | 1 | 14.5 | 26 | 3.3 | 21.9 | 0 | 9.1 | 8.5 | 19.1 | 1 | 66.53482 |
| 2020 | October | 10 | 15 | 2 | 14.5 | 26 | 3.3 | 21.9 | 0 | 9.1 | 8.5 | 19.1 | 1 | 49.85344 |
| 2020 | October | 10 | 15 | 3 | 14.5 | 26 | 3.3 | 21.9 | 0 | 9.1 | 8.5 | 19.1 | 1 | 46.3342 |
| 2020 | October | 10 | 15 | 4 | 14.5 | 26 | 3.3 | 21.9 | 0 | 9.1 | 8.5 | 19.1 | 1 | 44.94069 |
| 2020 | October | 10 | 15 | 5 | 14.5 | 26 | 3.3 | 21.9 | 0 | 9.1 | 8.5 | 19.1 | 1 | 42.84811 |
| 2020 | October | 10 | 15 | 6 | 14.5 | 26 | 3.3 | 21.9 | 0 | 9.1 | 8.5 | 19.1 | 1 | 40.74242 |
| 2020 | October | 10 | 15 | 7 | 14.5 | 26 | 3.3 | 21.9 | 0 | 9.1 | 8.5 | 19.1 | 1 | 39.42306 |
| 2020 | November | 11 | 15 | 1 | 9.1 | 18.7 | 0.3 | 50.3 | 17.01 | 7.5 | 7 | 14.8 | 1 | 51.43922 |
| 2020 | November | 11 | 15 | 2 | 9.1 | 18.7 | 0.3 | 50.3 | 17.01 | 7.5 | 7 | 14.8 | 1 | 32.11704 |
| 2020 | November | 11 | 15 | 3 | 9.1 | 18.7 | 0.3 | 50.3 | 17.01 | 7.5 | 7 | 14.8 | 1 | 30.69651 |
| 2020 | November | 11 | 15 | 4 | 9.1 | 18.7 | 0.3 | 50.3 | 17.01 | 7.5 | 7 | 14.8 | 1 | 33.94763 |
| 2020 | November | 11 | 15 | 5 | 9.1 | 18.7 | 0.3 | 50.3 | 17.01 | 7.5 | 7 | 14.8 | 1 | 35.4635 |
| 2020 | November | 11 | 15 | 6 | 9.1 | 18.7 | 0.3 | 50.3 | 17.01 | 7.5 | 7 | 14.8 | 1 | 33.85788 |
| 2020 | November | 11 | 15 | 7 | 9.1 | 18.7 | 0.3 | 50.3 | 17.01 | 7.5 | 7 | 14.8 | 1 | 29.87885 |
| 2020 | December | 12 | 15 | 1 | 3.3 | 11.4 | -2.8 | 75.7 | 28.44 | 5 | 6.8 | 15 | 1 | 96.52071 |
| 2020 | December | 12 | 15 | 2 | 3.3 | 11.4 | -2.8 | 75.7 | 28.44 | 5 | 6.8 | 15 | 1 | 77.0405 |
| 2020 | December | 12 | 15 | 3 | 3.3 | 11.4 | -2.8 | 75.7 | 28.44 | 5 | 6.8 | 15 | 1 | 73.28683 |
| 2020 | December | 12 | 15 | 4 | 3.3 | 11.4 | -2.8 | 75.7 | 28.44 | 5 | 6.8 | 15 | 1 | 75.58514 |
| 2020 | December | 12 | 15 | 5 | 3.3 | 11.4 | -2.8 | 75.7 | 28.44 | 5 | 6.8 | 15 | 1 | 78.02028 |
| 2020 | December | 12 | 15 | 6 | 3.3 | 11.4 | -2.8 | 75.7 | 28.44 | 5 | 6.8 | 15 | 1 | 77.50933 |
| 2020 | December | 12 | 15 | 7 | 3.3 | 11.4 | -2.8 | 75.7 | 28.44 | 5 | 6.8 | 15 | 1 | 74.00838 |
